# Nutrient shortage triggers the hexosamine biosynthetic pathway *via* the GCN2-ATF4 signalling pathway

**DOI:** 10.1038/srep27278

**Published:** 2016-06-03

**Authors:** Cédric Chaveroux, Carmen Sarcinelli, Virginie Barbet, Sofiane Belfeki, Audrey Barthelaix, Carole Ferraro-Peyret, Serge Lebecque, Toufic Renno, Alain Bruhat, Pierre Fafournoux, Serge N. Manié

**Affiliations:** 1 INSERM U1052, CNRS UMR5286, Centre de Recherche en Cancérologie de Lyon, F-69000 Lyon, France.; 2 Université de Lyon, Université Lyon 1, F-69000 Lyon, France; 3 Centre Léon Bérard, F-69008 Lyon, France; 4 INRA, UMR 1019 Nutrition Humaine, Centre de Clermont-Ferrand-Theix, 63122 Saint Genès Champanelle, France; 5 Université Clermont 1, UFR Médecine, UMR 1019 Nutrition Humaine, 63000 Clermont-Ferrand, France

## Abstract

The hexosamine biosynthetic pathway (HBP) is a nutrient-sensing metabolic pathway that produces the activated amino sugar UDP-N-acetylglucosamine, a critical substrate for protein glycosylation. Despite its biological significance, little is known about the regulation of HBP flux during nutrient limitation. Here, we report that amino acid or glucose shortage increase GFAT1 production, the first and rate-limiting enzyme of the HBP. GFAT1 is a transcriptional target of the activating transcription factor 4 (ATF4) induced by the GCN2-eIF2α signalling pathway. The increased production of GFAT1 stimulates HBP flux and results in an increase in O-linked β-N-acetylglucosamine protein modifications. Taken together, these findings demonstrate that ATF4 provides a link between nutritional stress and the HBP for the regulation of the O-GlcNAcylation-dependent cellular signalling.

The regulation of metabolic fluxes is a key process in the cellular response to changes in nutrient availability, and dysregulation of this process may contribute to the development of various diseases^1^,^2^. The hexosamine biosynthetic pathway (HBP) plays a central role in sensing the nutritional status of the cell, since it integrates molecules coming from carbohydrates, fatty acids, amino acids and nucleotides metabolism^3^. The HBP converts fructose-6-phosphate, a glucose derivative, into UDP-N-acetylglucosamine (UDP-GlcNAc), a central nucleotide sugar acting as a donor substrate for the glycosylation of proteins and lipids. In addition, UDP-GlcNAc is used for the O-GlcNAcylation of proteins, i.e. the addition of N-acetylglucosamine to their serine and threonine residues. Similarly to phosphorylation, O-GlcNAcylation regulates cellular activities, such as signalling and transcription^4^. It has been proposed that O-GlcNAcylated proteins modulate cellular responses to nutrient deprivation and stress, and that their dysregulation is implicated in a wide range of pathologies^5^. Despite these wide-ranging biological effects, the mechanisms underlying the adaptation of the HBP flux to nutrient availability remains poorly understood.

The first and rate-limiting enzyme of the HBP, namely glutamine:fructose-6-phosphate aminotransferase 1 (GFAT1), which drives the HBP flux, is involved in various physiopathological processes^6^,^7^,^8^,^9^. GFAT1 activity is inhibited by UDP-GlcNAc, the end product of the HBP^10^, and the AMP-activated protein kinase-mediated phosphorylation^11^. In contrary, GFAT1 is stimulated upon phosphorylation by protein kinase A and calcium/calmodulin-dependent protein kinase II^12^. More recently, it was shown that GFAT1 production is upregulated by a transcription factor of the unfolded protein response (UPR), namely the spliced form of X-box binding protein 1 (XBP1s), resulting in the stimulation of the HBP flux and protein O-GlcNAcylation^13^. These authors unveiled in this way an intrinsic link between the HBP and disruption of the endoplasmic reticulum (ER) homeostasis, i.e. ER stress, which activates the UPR.

UPR signalling is mediated by three ER transmembrane sensors: IRE1α (Inositol requiring enzyme 1α), PERK (PKR-like Endoplasmic Reticulum kinase), and ATF6α (activating transcription factor 6α)^14^. Activation of the UPR induces global changes in gene expression to restore ER homeostasis, and can trigger apoptosis when the ER stress cannot be alleviated. Although XBP1s, controlled by IRE1α, directly promotes the transcription of GFAT1^13^, high throughput data suggest that the activating transcription factor 4 (ATF4), another UPR effector controlled by the PERK-mediated phosphorylation of the α subunit of the eukaryotic initiation factor 2 (eIF2α), also regulates GFAT1 expression^15^,^16^. This finding was of particular interest since eIF2α phosphorylation represents an integrator of various cellular cues and therefore occurs not only during ER stress. Indeed, in addition to PERK, three other kinases converge to phosphorylate eIF2α, namely HRI (heme regulated inhibitor) that responds to heme deprivation, GCN2 (general control nonderepressible 2) that responds to amino acid deprivation, and PKR (protein kinase RNA-activated), which is activated by double-stranded RNA^17^. Because of the convergence of these kinases in the phosphorylation of eIF2α, the eIF2α-ATF4 pathway is often referred to as the integrated stress response pathway (ISR). This pathway leads to a downregulation of the translation of most proteins, but concomitantly, to an upregulation in the translation of specific transcripts with open reading frames (uORFs) in their 5′ untranslated region. One of these transcripts, ATF4 has been extensively characterized; it controls a transcriptional program of genes involved in multiple processes, such as amino acid and lipid homeostases, and redox balance^17^.

Here, we report that GFAT1 is a direct target of ATF4. We demonstrate that amino acid or glucose shortage increase GFAT1 production through the GCN2-eIF2α-ATF4 pathway, which results in the stimulation of protein O-GlcNAcylation. Our investigation deciphers novel mechanisms underlying the regulation of GFAT1, and reveals that an increase in O-GlcNAcylated proteins occurs during not only glucose shortage but also amino acid deprivation.

## Results

### GFAT1 is a direct transcriptional target for ATF4.

**Figure 1:**
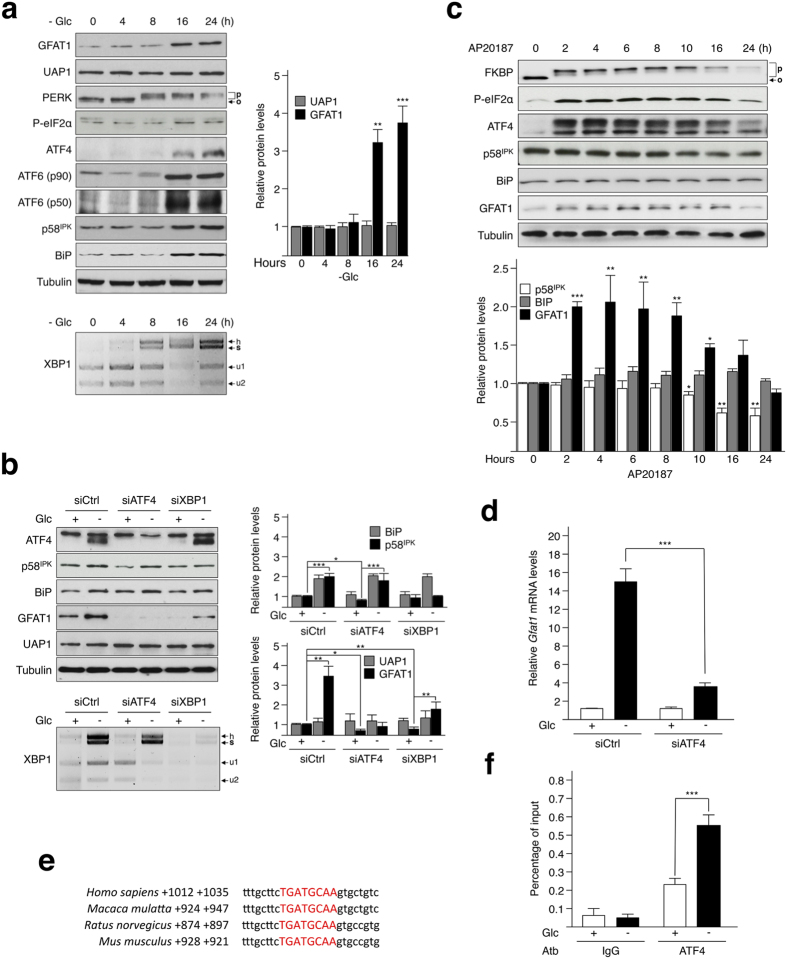
ATF4 positively controls the expression of GFAT1. **(a)** Top: Western blot analysis of UPR markers and HBP enzymes upon Glc shortage (0.1 mM) for the indicated times (h = hours). O, inactivated and P, activated PERK. Quantification of proteins bands of independent experiments, compared to 0 h condition and normalized to the loading control tubulin is shown. Bottom: *XBP1* mRNA splicing was analysed by RT-PCR followed by Pst1 digestion: u1 and u2, unspliced and s, spliced variant of *XBP1;* h, hybrid between unspliced and spliced ssDNA. **(b)** Top: Cells transfected either with control siRNA (Ctrl) or siRNA against ATF4 or XBP1 were incubated with 25 mM Glc (+) or 0.1 mM Glc (−) for 24 hours and analyzed by Western blotting with the indicated antibodies. Quantification of proteins bands of independent experiments and normalized to the loading control tubulin is shown. Bottom: *XBP1* mRNA splicing was analysed by RT-PCR as described in a. (**c)** Rat1 cells stably expressing the Fv2E-PERK construct and treated with 2 nM AP20187 over a time course were analyzed by Western blotting with the indicated antibodies. O, inactivated and P, activated Fv2E-PERK. Quantification of proteins bands was performed as in a. (**d)** RT-qPCR analysis of *Gfat1* mRNA isolated from cells transfected with a control siRNA (Ctrl) or an ATF4 siRNA and incubated for 16 hours with 25 mM Glc (+) or 0.1 mM Glc (−). Data were normalized against endogenous *Hprt* mRNA levels. **(e)** Distance of a conserved putative-ATF4 binding site from the *Gfat1* gene found in several species. **(f)** ChIP analysis of ATF4 binding to ATF4 binding site shown in e. Cells were incubated for 16 hours with 25 mM Glc (+) or 0.1 mM Glc (−). An irrelevant IgG antibody serves as a control. For blots shown in a, b and c, samples derive from the same experiment and gels/blots were processed in parallel. Full-length blots/gels are presented in Supplementary Figure S7. *p < 0.05, **p < 0.01, ***p < 0.001.

The HBP processes 2–5% of the glucose (Glc) metabolism in cells. When Glc becomes scarce, a paradoxical increase in HBP flux occurs. Previous experiments demonstrated the role of UPR in increasing GFTA1 and HBP flux during starvation, i.e. medium free of glucose, serum, pyruvate and glutamine^13^. Here, we found that limiting the amount of Glc (0.1 mM) in the growth medium was sufficient to increase GFAT1 protein levels in transformed human bronchial epithelial cells (HBEC 3KT-RL)^18^. Glucose starvation led to the activation of the three branches of the UPR after 8 hours of incubation (Fig. 1a). This activation was detected by monitoring (i) the splicing of XBP1 mRNA, a key marker of the IRE1α branch^19^, (ii) the cleavage of ATF6 that releases its 50-kDa b-ZIP transcription factor^20^, (iii) the abundance in the BiP protein, which is mainly controlled by the activation of the ATF6 branch^21^, (iv) the abundance in p58^IPK^ that is regulated both by the ATF6 and IRE1α branches^21^, and (v) the level of phosphorylated PERK and eIF2α, which in turn controls the expression of ATF4^22^. Furthermore, the production of GFAT1, but not that of UAP1 another enzyme of the HBP, increased at 16 and 24 hours following Glc shortage (Fig. 1a). Pharmacological activation of the UPR using tunicamycin also increased GFAT1 production (Supplementary Fig. S1). Corroborating a role for XBP1s in the regulation of GFAT1, its silencing reduced GFAT1 production (Fig. 1b)^13^. However, we show that GFAT1 production is not solely linked with the XBP1 branch of the UPR, since the extinction of ATF4 by siRNA also prevented GFAT1 production. This latter result was not due to the nonspecific silencing of the IRE1 pathway following the transfection with ATF4 siRNA, since both the splicing of XBP1 mRNA and the increased in p58^IPK^ abundance were barely affected by this transfection (Fig. 1b). Of note, the production of UAP1 was not altered following transfection with either XBP1 or ATF4 siRNAs, whereas the basal protein level of GFAT1 in HBEC 3KT-RL cells decreased under the same conditions.

To further investigate the specific effect of ATF4 on GFAT1 abundance, we turned to a cellular model of Rat1 embryonic fibroblasts stably expressing Fv2E-PERK. Indeed, this construct produces a chimeric PERK kinase lacking the ER luminal domain and fused with two modified FK506 binding domains (FKBP). This system enables, through the inducible activation of the Fv2E-PERK fusion protein by the dimerizing drug AP20187^23^, the monitoring of PERK signalling in an ER stress-independent manner, i.e. in the absence of IRE1 and ATF6 activation. Treatment of Rat1 cells with AP20187 induced a strong activation of Fv2E-PERK (Fig. 1c), as attested by the phosphorylation-induced shift in the migration of FKBP^23^, which resulted in an increase in eIF2α phosphorylation and ATF4 production, as well as in GFAT1 abundance. As expected, protein abundance of two main XBP1s and ATF6 targets, respectively p58^IPK^ and BiP, was not affected upon AP20187 treatment. These results, thus, show that the sole activation of the PERK-ATF4 axis is sufficient to increased GFAT1 abundance.

Next, we examined whether GFAT1 is a direct target of ATF4 following nutrient depletion. GFAT1 mRNA production following glucose shortage was strongly reduced in HBEC 3KT-RL cells upon silencing of ATF4, indicating that the effect of ATF4 likely occurs at the transcriptional level (Fig. 1d). Furthermore, no differences in the basal transcription level of GFAT1 (i.e. Glc+) were observed in the ATF4-silenced cells, in contrast to the marked reduction in GFAT1 protein levels observed in the Western blot analysis (Fig. 1b). This suggests that ATF4-dependent regulation of GFAT1 at steady state may occur post-transcriptionally. We then studied the binding of ATF4 to the *Gfat1* DNA sequence upon glucose shortage, using chromatin immunoprecipitation (ChIP) assays on a cross-species conserved ATF4-binding site, identified by previous ATF4 ChIP-sequencing experiments^16^ and located close to the *Gfat1* gene (Fig. 1e). ChIP experiments confirmed that ATF4 recruitment to this DNA sequence was increased during glucose deprivation, indicating that GFAT1 is a direct transcriptional target of ATF4 (Fig. 1f). Hence, together with high throughput data^15^,^16^, these results indicate that GFAT1 is regulated at the transcriptional level by ATF4.

### Activation of the eIF2α-ATF4 pathway stimulates protein O-GlcNAcylation

**Figure 2:**
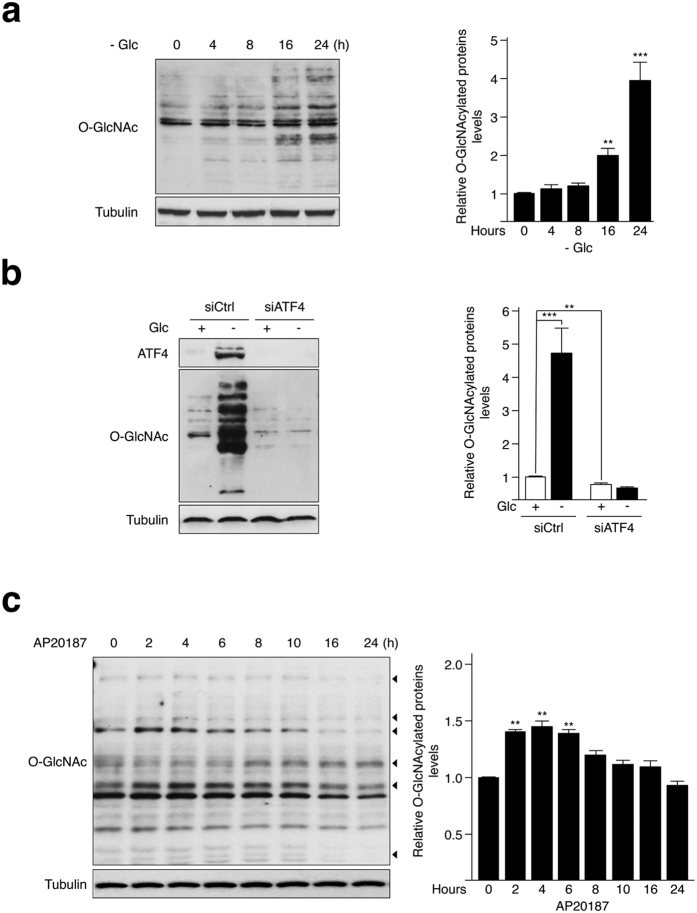
Activation of the eIF2α-ATF4 signalling pathway enhances the flux of the HBP upon glucose deprivation. **(a)** HBEC 3KT-RL cells incubated with 0.1 mM Glc over a time course of 24 hours were analyzed by Western blot developed with antibody against O-GlcNAc-modified proteins. Quantification of the intensity of O-GlcNAcylated protein bands of independent experiments, compared to 0 h condition and normalized to the loading control tubulin is shown. **(b)** Western blot analysis of O-GlcNAcylated proteins and ATF4 protein abundance in cells transfected with a control siRNA (siCtrl) or an ATF4 siRNA and incubated with 25 mM Glc (+) or 0.1 mM Glc (−) for 24 hours. Samples derive from the same experiment and gels/blots were processed in parallel. Quantification of the intensity of O-GlcNAcylated protein bands of of independent experiments and normalized to the loading control tubulin is shown. **(c)** Rat1 cells stably expressing the Fv2E-PERK construct and treated with 2 nM AP20187 over a time course were analyzed by Western blot developed with antibody against O-GlcNAc-modified proteins. Arrowheads exemplify bands with the substantial changes in O-GlcNAcylation levels. Quantification of the intensity of O-GlcNAcylated protein bands of of independent experiments, compared to 0 h condition and normalized to the loading control tubulin is shown. Full-length blots/gels are presented in Supplementary Figure S7. **p < 0.01, ***p < 0.001.

GFAT1 is the first and rate-limiting enzyme of the HBP, which produces UDP-GlcNAc and consequently drives cellular O-GlcNAcylation. To determine whether the ATF4-GFAT1 pathway we identified here affects the HBP flux, we monitored the level of cellular O-GlcNAcylation by Western blotting. Glucose shortage increased O-GlcNAcylation in HBEC 3KT-RL (Fig. 2a), consistently with a previous report^24^. This increase was strongly correlated with the augmentation in GFAT1 abundance observed in Fig. 1a and a siRNA approach confirmed the key role for this enzyme in driving O-GlcNAcylation during glucose deprivation (Supplementary Fig. S2). In addition, the silencing of ATF4 prevented Glc deprivation-induced O-GlcNAcylation (Fig. 2b), indicating that ATF4-mediated GFAT1 control is biologically relevant. It is of note that O-GlcNAcase (OGA) and O-GlcNAc transferase (OGT) protein abundance was not affected by ATF4 silencing (Supplementary Fig. S3). Conversely, the pharmacological activation of the PERK-ATF4 pathway in Rat1 cells using AP20187, resulted in an increase in O-GlcNAcylation of various protein bands over the time course of the experiment (Fig. 2c). Thus, ATF4-mediated control of GFAT1 abundance stimulates protein O-GlcNAcylation.

### GCN2 stimulates the hexosamine biosynthesis pathway upon either glucose or amino acid shortage

**Figure 3:**
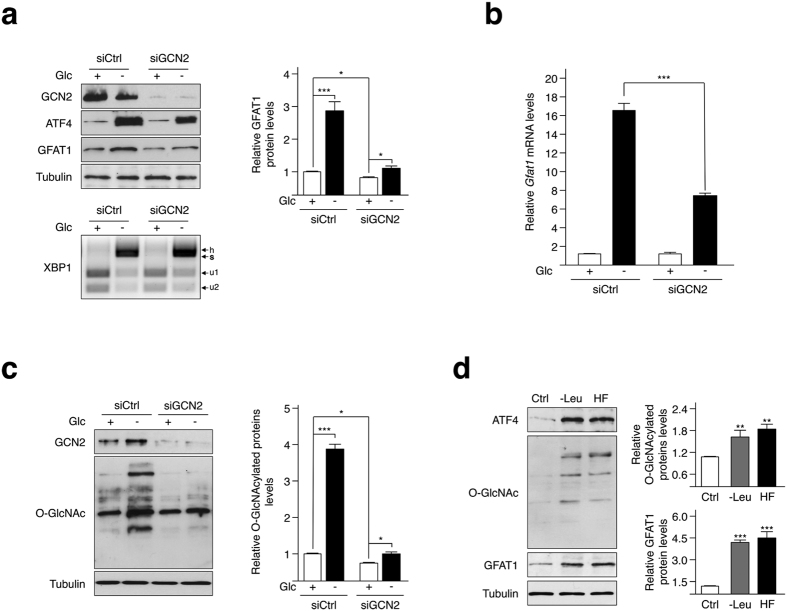
GCN2 participates in the ATF4-dependent regulation of protein O-GlcNAcylation. **(a)** Cells transfected with a control (siCtrl) or GCN2 siRNA, and incubated with 25 mM Glc (+) or 0.1 mM Glc (−) for 24 hours, were analyzed by Western blotting with the indicated antibodies. Quantification of proteins bands of independent experiments, compared to 0 h condition and normalized to the loading control tubulin is shown. Bottom: *XBP1* mRNA splicing was analysed by RT-PCR followed by Pst1 digestion: u1 and u2, unspliced and s, spliced variant of *XBP1;* h, hybrid between unspliced and spliced ssDNA. **(b)** RT-qPCR analysis of *Gfat1* mRNA isolated from cells transfected with a control siRNA (Ctrl) or a GCN2 siRNA and incubated for 16 hours with 25 mM Glc (+) or 0.1 mM Glc (−). Data were normalized against endogenous *Hprt* mRNA levels. **(c)** Western blot analysis of O-GlcNAcylated proteins and GCN2 protein abundance in cells transfected with a control siRNA (siCtrl) or a GCN2 siRNA and incubated with 25 mM Glc (+) or 0.1 mM Glc (−) for 24 hours. Quantification of the intensity of O-GlcNAcylated protein bands of of independent experiments and normalized to the loading control tubulin is shown. **(d)** Cells deprived of leucine (-Leu) or treated with 100 nM of halofuginone (HF) for 16 hours were analyzed by Western blotting with the indicated antibodies. Quantification of the intensity of O-GlcNAcylated protein bands of independent experiments, compared to normal medium containing leucine condition (control, Ctrl) and normalized to the loading control tubulin is shown. For blots shown in a, c and d, samples derive from the same experiment and gels/blots were processed in parallel. Full-length blots/gels are presented in Supplementary Figure S7. *p < 0.05, **p < 0.01, ***p < 0.001.

**Figure 4:**
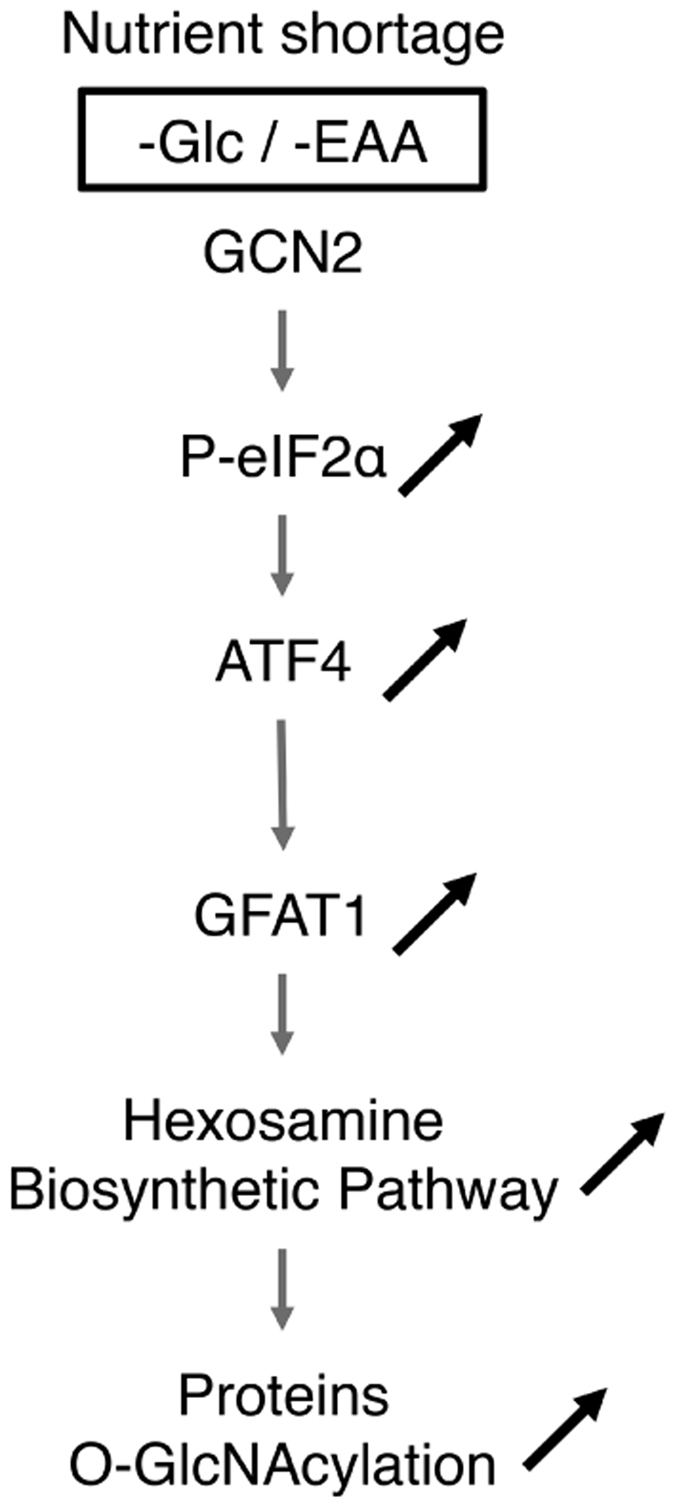
Schematic representation highlighting the molecular link between the GCN2 pathway and protein O-GlcNAcylation in nutrients-deprived cells. Following glucose or essential amino acid shortage, activation of the GCN2-eIF2α-ATF4 stress pathway increases GFAT1 production. Consequently, the stimulation of the flux of the HBP contributes to increased protein O-GlcNAcylation.

Having explored the activation of the HBP via the PERK-dependent activation of the ISR, we reasoned that another eIF2α kinase, namely GCN2, could also modulate O-GlcNAcylation. Indeed, GCN2 is a well-described sensor for amino acid availability and recent reports have also described that it can be activated in response to glucose starvation^25^,^26^,^27^. We found that GCN2 silencing in HBEC 3KT-RL cells moderated Glc shortage-induced ATF4 upregulation (Fig. 3a), and that similar results were obtained in GCN2-deficient MEFs (Supplementary Fig. S4a). We then focused our investigations on a stable cell line expressing the luciferase reporter gene under the control of CARE sequences of the *Trib3* promoter, which was previously described to reflect the transcriptional activity of ATF4^28^. Using this system, we observed that silencing of GCN2 reduced the luciferase activity by half in response to glucose starvation that correlated with a reduced induction of ATF4 target genes (Supplementary Fig. S4b,c). Thus, these results are consistent with the fact that GCN2 activation contributes to the increase in ATF4 abundance when cells are facing glucose limitation. In line with these results, GCN2 silencing prevented the Glc shortage-induced GFAT1 upregulation both at the mRNA and protein levels (Fig. 3b,a, respectively), and also lowered the level of cellular O-GlcNAcylation (Fig. 3c). Interestingly, Glc shortage induced an increase in OGT protein expression that could be prevented by GCN2 silencing (Supplementary Fig. S5), but not by ATF4 silencing as mentioned above. Hence, GCN2 activation may further increase protein O-GlcNAcylation in an ATF4-independent manner, by stimulating OGT production through yet unclear mechanisms. Finally, we tested whether GCN2 activation following leucine deprivation^29^ or treatment with halofuginone, a pharmacological inducer of GCN2^30^, also stimulates O-GlcNAcylation (Fig. 3d). As expected, both treatments conducted for 16 hours resulted in an increase in ATF4 and GFAT1 abundance, and correspondingly promoted O-GlcNAcylation in HBEC 3KT-RL cells. Hence, our findings demonstrate a molecular connection between the kinase GCN2 and the hexosamine biosynthetic pathway, and that augmentation of O-GlcNAcylated proteins occurs in the context of glucose deprivation and during amino acid shortage as well (Fig. 4).

## Discussion

The hexosamine biosynthetic pathway is involved in the post-translational addition of O-GlcNAc onto proteins that regulates gene transcription and cell signalling^4^. Remarkably, the HBP flux increases during glucose shortage. Here we show that this up-regulation occurs not only during glucose shortage but also during amino acid deprivation. In both situations, the GCN2-mediated phosphorylation of eIF2α and the subsequent ATF4-driven expression of GFAT1 drive proteins O-GlcNAcylation. Also, we reveal that GFAT1 production controlling protein O-GlcNAcylation can occur independently of the previously described role of XBP1s during UPR^13^.

We unveil a novel role for ATF4, beyond its function during cellular stress responses. Indeed, when grown under glucose-rich medium (25 mM Glc), basal levels of ATF4 and XBP1s were detected in HBEC 3KT-RL cells. Following ATF4 and XBP1 silencing, the GFAT1 protein was no longer detected, while *Gfat1* transcripts were not affected. In support of a specific ATF4 and XBP1s-mediated maintenance of GFAT1 abundance at steady state, the basal level of the HBP enzyme PGM3 was not affected, whereas XBP1s silencing prevented glucose shortage-induced PGM3 up-regulation as previously reported^13^ (Supplementary Fig. S6). These findings are consistent with an emerging role for the UPR in buffering normal cellular fluctuations, in order to maintain the physiological functions of the cell^31^. Further studies are required to clarify whether an UPR-dependent post-translational mechanism is responsible for this basal regulation.

Our investigations also uncovered that amino acid deprivation and glucose shortage modulated GFAT1 production, via a GCN2-dependent signalling pathway. As noted previously, we initially confirmed that the GCN2 pathway responded to glucose starvation^25^,^26^,^27^, as well as to conventional amino acid depletion. We then demonstrated that this pathway contributed to ATF4 protein production and to the transcription of ATF4-dependent genes (Supplementary Fig. S4a–c). A recent functional study conducted by Shin *et al.* reported the sequential activation of PERK and GCN2 during glucose limitation^27^. Indeed, they revealed that PERK activation was an early event (within 18 hours), whereas GCN2 activation occurred at a later stage (54 hours), and triggered cell death^27^. In our study, PERK and GCN2 activation occurred almost concomitantly. This simultaneous activation may be linked to the glucose concentration in the growth medium, rather than being a time-dependent event, since Shin *et al.* noted GCN2 activation once glucose concentration had dropped below ∼0.2 mM, whereas in our study, cells are grown in 0.1 mM glucose from the start. Thus, GCN2 activation likely occurred earlier under our experimental setting and contributed to ATF4-dependent transcription in parallel to the reported role of PERK^27^.

Owing to the fact that the GCN2 activation is associated with amino acid depletion, how GCN2 responds to glucose deprivation remains largely unknown. This kinase senses the accumulation of uncharged tRNAs upon amino acid starvation, which in turn stimulates its catalytic domain, and leads to the subsequent phosphorylation of eIF2α. Wellen *et al.* showed that the uptake in glutamine and leucine diminishes upon glucose shortage, since the level of amino acid transporter mRNA is reduced^32^. However, this hypothesis seems not to be compatible with the relatively rapid mobilization of the GCN2 pathway observed in the present study (<8 hours, Supplementary Fig. S4a,c). Notably, it is becoming evident that the GCN2 pathway is implicated in other biological processes that are apparently unrelated to amino acid starvation. However, mechanisms underlying the integration of these activating signals are far from being understood^33^. In the case of glucose withdrawal, previous studies have described a rapid (<3 hours) induction of reactive oxygen species (ROS)^34^. Given that GCN2 is activated by ROS^35^,^36^, it is tempting to speculate that there is a connection between glucose deprivation, ROS generation and GCN2 activation. Further studies are required to verify this hypothesis.

Although GCN2 activation may induce cell death upon severe glucose shortage^27^, it is also conceivable that GCN2-mediated HBP stimulation might have pro-survival functions in the case of moderate glucose shortage. Indeed, it appears that O-GlcNAcylation has most often a pro-survival function, particularly in cardiomyocytes subjected to ischemia/reperfusion^13^ or in cancer cells^37^,^38^. The HBP flux regulates the expression of growth factor receptors at the surface of cells, which controls glutamine and leucine uptake^32^. Glutamine, like glucose, is used by proliferating cells to fuel a variety of bioenergetic and biosynthetic pathways^39^. GCN2-mediated HBP stimulation may favor the uptake of glutamine to enable cells to compensate at least partly and/or momentarily for the lack of glucose.

In summary, we report that the GCN2-ATF4 pathway links amino acid and glucose starvation to protein O-GlcNAcylation by stimulating the HBP flux. Given the recognition of the importance of O-GlcNAcylation in cell signalling, our findings reveal novel biological functions for GCN2 that may eventually lead to the development of therapeutic strategies against diseases related to HBP dysregulation.

## Methods

### Cell culture, treatments and nutrient deprivation experiments

The cell lines used in this study were either generated in-house in the case of the Fv2E-PERK Rat-1 and CARE-Luc HeLa cells, or donated by Drs. D. Ron^40^ and J.D. Minna^41^ in the case of the wild type Mouse Embryonic Fibroblasts (MEFs) or general control nonderepressible 2 (GCN2) deficient MEFs, and Human Bronchial Epithelial Cells (HBEC 3KT-RL), respectively. The cell lines were grown in Dulbecco’s High Glucose Modified Eagle’s Medium (DMEM) supplemented with 10% fetal bovine serum, sodium pyruvate, L-glutamine and antibiotics, except for the HBEC 3KT-RL cells, which were grown in Keratinocyte Serum-Free Medium (KSFM) supplemented with bovine pituitary extract and recombinant human EGF. The cells were maintained at 37 °C with 5% CO_2_ throughout the experiments.

For glucose deprivation experiments, cells were rinsed with Phosphate-Buffered Saline (PBS) and incubated in DMEM containing either 25 mM glucose for the control cells, 0.1 mM glucose for HBEC 3KT-RL glucose-starved cells, or no glucose for MEFs and HeLa glucose-deprived cells. Glucose deprivation assays were carried out over a time course of 24 hours (0, 4, 8, 16 or 24 hours). Leucine starvation experiments were conducted over 16 hours using a DMEM/Nutrient Mixture: F-12 (DMEM/F12) medium devoid of leucine, glutamine, lysine and methionine (Sigma-Aldrich, ref: D9785), in which glutamine, lysine, methionine and 10% dialyzed serum were subsequently added. The control medium for these experiments was similar to that used previously but supplemented with leucine. Activation of the Fv2E-PERK fusion protein was conducted by treating cells with 2 nM AP20187 (Clonetech, ref: 635060). The pharmacological activation of the GCN2 pathway was achieved by treating cells with 100 nM of halofuginone (Sigma-Aldrich) for 16 hours. Experiments were repeated at least three times. The chemical induction of the ER stress pathways was performed by adding 0.5 μg/mL of tunicamycin (Sigma-Aldrich, ref: T7765) to the culture medium. An equivalent volume of DMSO was used for control experiments.

### Lipid-mediated siRNA delivery

HBEC 3KT-RL cells were transfected with either known human siRNAs in the case of GCN2 (sc-45644), ATF4 (sc-35112), XBP1 (sc-38627) and scrambled siRNA (sc-37007) (Santa Cruz Biotechnology), or with in-house designed human GFAT1 siRNA. Indeed, the forward and reverse primer sequences (Supplementary Table 1) were obtained based on data from the RNAi consortium (TRC), the siRNA duplex was then synthesized by Eurogentec. Briefly, cells were transfected in 6-well plates (typically 400,000 cells per well) using a combination of 25 ρM of siRNA duplex and 12.5 μL of HiPerFect reagent (Qiagen) according to the manufacturer’s reverse transfection protocol. Cells were treated or lysed 72 hours post-transfection.

### Gene expression profiling and XBP1 splicing assessment

Following total RNA extraction from HBEC 3KT-RL cells using the Nucleospin RNA kit (Macherey-Nagel), 0.5 μg of RNA were reverse transcribed using random primers according to the instructions supplied with the Superscript II RNase H Reverse Transcriptase kit (Invitrogen). The cDNA was then amplified by qPCR using primers specific for the *Chop, Gadd34, Gfat1* and *Trib3* genes*, Hprt* serving as a control (see Supplementary Table S1), using the Kapa SYBR Fast qPCR kit (KapaBiosystems) and an ABI prism instrument (AppliedBiosystems). QPCR experiments were repeated at least three times with three replicates per group.

The active form of XBP1 is its spliced form. The unspliced form of XBP1 possesses a Pst1 restriction site, which is not present on the spliced form. The level of spliced XBP1 was assessed by amplifying its cDNA using the primer pair provided in the Supplementary Table S1 and performing a subsequent enzymatic digestion targeting the Pst1 site. The digest was then run on a 2.5% agarose gel. The inactive/unspliced form resulted in two small fragments (u1 and u2), following the restriction digest of Pst1^42^,^43^, while the active/spliced form (bold s) remained undigested. A fourth band was also obtained and corresponded to a hybrid (h) between the unspliced and spliced ssDNA formed during the PCR.

### Western blotting

The primary antibodies used during the Western blot analyses were purchased either from Santa Cruz Biotechnology: ATF4 (sc-200); from Cell Signaling Technology: GCN2 (3302), PERK (3192), P-eIF2α (3398), CHOP (2895) and p58^IPK^ (2940); from Sigma-Aldrich: α-Tubulin (T6199), OGT (HPA030751), OGA (SAB4200311) and O-GlcNAc (clone CTD110.6, O7764); from Abcam: ATF6 (ab122897), UAP1 (ab155287), PGM3 (ab128094) and GFAT1 (ab125069); from BD Biosciences: BiP (610978), or from Enzo life sciences: FKBP (ALX-210-142). The secondary anti-rabbit or anti-mouse antibodies were supplied by Jackson Laboratories.

Briefly, whole cell extracts were obtained from cultured cells using a RIPA protein buffer containing protease and phosphatase inhibitors (Roche) and PUGNAc (Sigma-Aldrich) and then quantified using the DC protein assay (Bio-Rad). Proteins (20 μg) were separated by SDS-PAGE, transferred onto nitrocellulose membranes and blocked for 1 hour at room temperature in Tris-Buffered Saline and Tween 20 (TBST) containing either 5% milk or Bovine Serum Albumin (BSA) for O-GlcNAc analyses. Membranes were incubated overnight with the relevant primary antibodies, washed three times in TBST, and were incubated for 1 hour with the relevant secondary antibodies diluted in TBST containing 5% milk. After washing three times with TBST, proteins were detected using the Clarity western ECL substrate (Bio-Rad). Representative Western blot was shown and proteins bands quantification of three independent experiments was performed using the ImageJ software.

### Luciferase assays

For luciferase assays, the CARE-Luc cell line expressing the firefly luciferase reporter gene under the control of CARE sequences was used^44^,^15^,^45^. Cells (5 × 10^5^) were plated in 12-well plates and transfected for 72 hours with scrambled or GCN2 siRNAs as described above. Cells were subsequently glucose-starved for 16 hours and then collected in 100 μL of lysis buffer (Promega). After centrifugation at 13,000 RPM for 2 min, 2 μL of the supernatant were assayed for luciferase activity using the Dual-luciferase reporter assay system (Promega). Measured luciferase activities were normalized against protein content (relative light units per milligram of protein). Luciferase assays were repeated at least three times with three replicates per group.

### Chromatin immunoprecipitation analysis (ChIP)

The antibodies used during the ChIP assays, namely the ATF4 rabbit polyclonal antibody (sc-200X) and normal rabbit IgG (sc-2027), were purchased from Santa Cruz Biotechnology (Santa Cruz, CA). HBEC 3KT-RL cells (1.5 × 10^6^) were seeded onto 150 mm in diameter dishes, 3 dishes being used per treatment. ChIP analyses were performed according to a previously published protocol^46^. DNA enrichment was analyzed by qPCR using the primer sequences listed alongside the genomic location of the amplified region in the Supplementary Table S1. All experiments were performed in triplicate to ensure reproducibility. The results are presented as ratio of precipitated DNA to input DNA.

### Bioinformatics alignments and statistical analyses

The ATF4-binding site, previously identified using the high throughput method, was aligned with the human genome using the UCSC genome browser (https://genome.ucsc.edu). The comparative alignment of DNA sequences across several species was performed using the Multiz Alignment tool and was confirmed using ClustalX (www.clustal.org).

The Student’s t-test or the one-way ANOVA and Tuckey post-hoc test were applied to determine the statistical differences between two or four experimental groups, respectively. All data are expressed as means ± SEM. *p < 0.05, **p < 0.01, ***p < 0.001.

## Additional Information

**How to cite this article**: Chaveroux, C. *et al.* Nutrient shortage triggers the hexosamine biosynthetic pathway *via* the GCN2-ATF4 signalling pathway. *Sci. Rep.* **6**, 27278; doi: 10.1038/srep27278 (2016).

## Supplementary Material

Supplementary Information
