# Supplementary material for: Nutrient shortage triggers the hexosamine biosynthetic pathway via the GCN2-ATF4 signalling pathway
Source: Sci Rep. 2016 Jun 3;6:27278. doi: 10.1038/srep27278 (PMC4891703; doi:10.1038/srep27278)
Supplement: Supplementary Information [file srep27278-s1.pdf]

Supplementary information

Nutrient shortage triggers the hexosamine biosynthetic pathway *via* the GCN2-ATF4 signalling pathway.

Cédric Chaveroux, Carmen Sarcinelli, Virginie Barbet, Sofiane Belfeki, Audrey Barthelaix, Carole Ferraro-Peyret, Serge Lebecque, Toufic Renno, Alain Bruhat, Pierre Fafournoux and Serge N Manié*

*Correspondence: serge.manie@lyon.unicancer.fr

The PDF file includes:

- Supplementary Figure S1: Tunicamycin treatment increases GFAT1 abundance in HBEC 3KT-RL cells.
- Supplementary Figure S2: GFAT1 silencing impairs proteins O-GlcNAcylation in glucose-deprived cells.
- Supplementary Figure S3: OGT and OGA protein analysis in HBEC 3KT-RL cells silenced for ATF4 and glucose-deprived for 24 hours.
- Supplementary Figure S4: GCN2 contributes to the ATF4-mediated transcription in response to glucose deprivation.
- Supplementary Figure S5: OGT and OGA expression levels in HBEC 3KT-RL cells silenced for GCN2 and glucose-deprived for 24 hours.
- Supplementary Figure S6: PGM3 abundance in HBEC 3KT-RL cells silenced for ATF4 or XBP1 and glucose-deprived for 24 hours.
- Supplementary Figure S7: Uncropped blots corresponding to main Figures 1, 2, and 3.
- Supplementary Table S1: Primers sequences used for RT-PCR, RT-qPCR, ChIP experiments and GFAT1 siRNA.

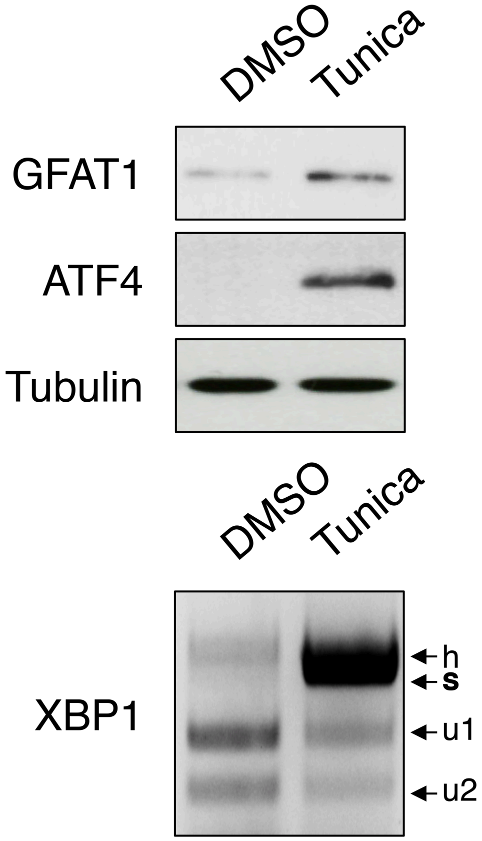

Supplementary Figure S1: Tunicamycin treatment increases GFAT1 abundance in HBEC 3KT-RL cells. Immunoblot analysis of the abundance of GFAT1 and ATF4 from HBEC 3KT-RL cells treated with DMSO or tunicamycin (0.5 µg/mL) for 16 hours. Tubulin serves as a loading control. Corresponding mRNAs were analyzed to assess XBP1 splicing by RT-PCR followed by Pst1 restriction: bold s- spliced XBP1, h- hydrid XBP1, u1 and u2- products from Pst1 cleavage of unspliced XBP1.

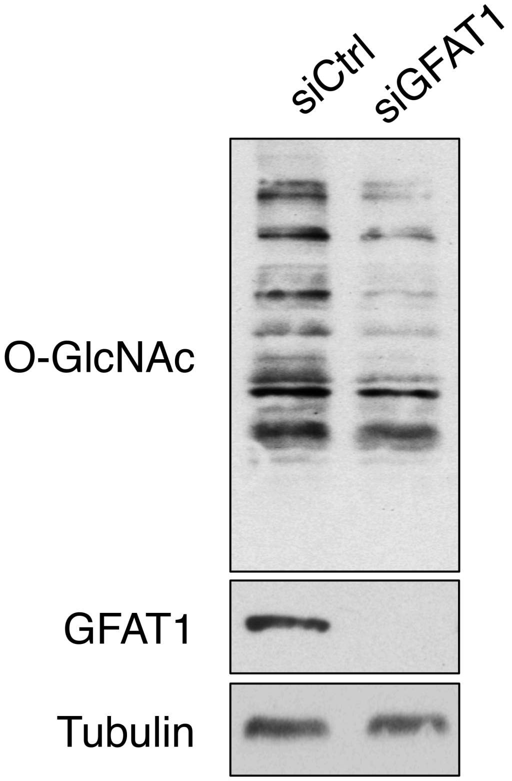

Supplementary Figure S2: GFAT1 silencing impairs proteins O-GlcNAcylation in glucose-deprived cells. Immunoblot analysis of the abundance of O-GlcNAcylated proteins and GFAT1 from HBEC 3KT-RL transfected with a control siRNA or GFAT1 siRNA and incubated in a medium containing 0.1 mM of glucose for 24 hours. Tubulin serves as a loading control.

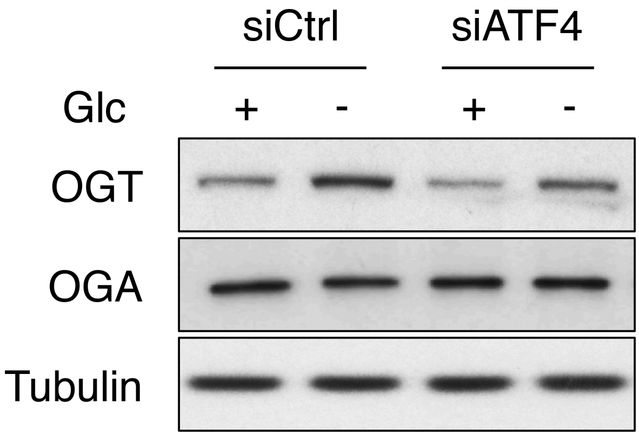

Supplementary Figure S3: OGT and OGA protein analysis in HBEC 3KT-RL cells silenced for ATF4 and glucose-deprived for 24 hours. Immunoblot analysis of the abundance of OGT and OGA, from HBEC 3KT-RL cells transfected with a control siRNA or an ATF4 siRNA. Cells were incubated with DMEM containing 25 mM (+) or 0.1 mM (-) of glucose for 24 hours. Tubulin serves as a loading control.

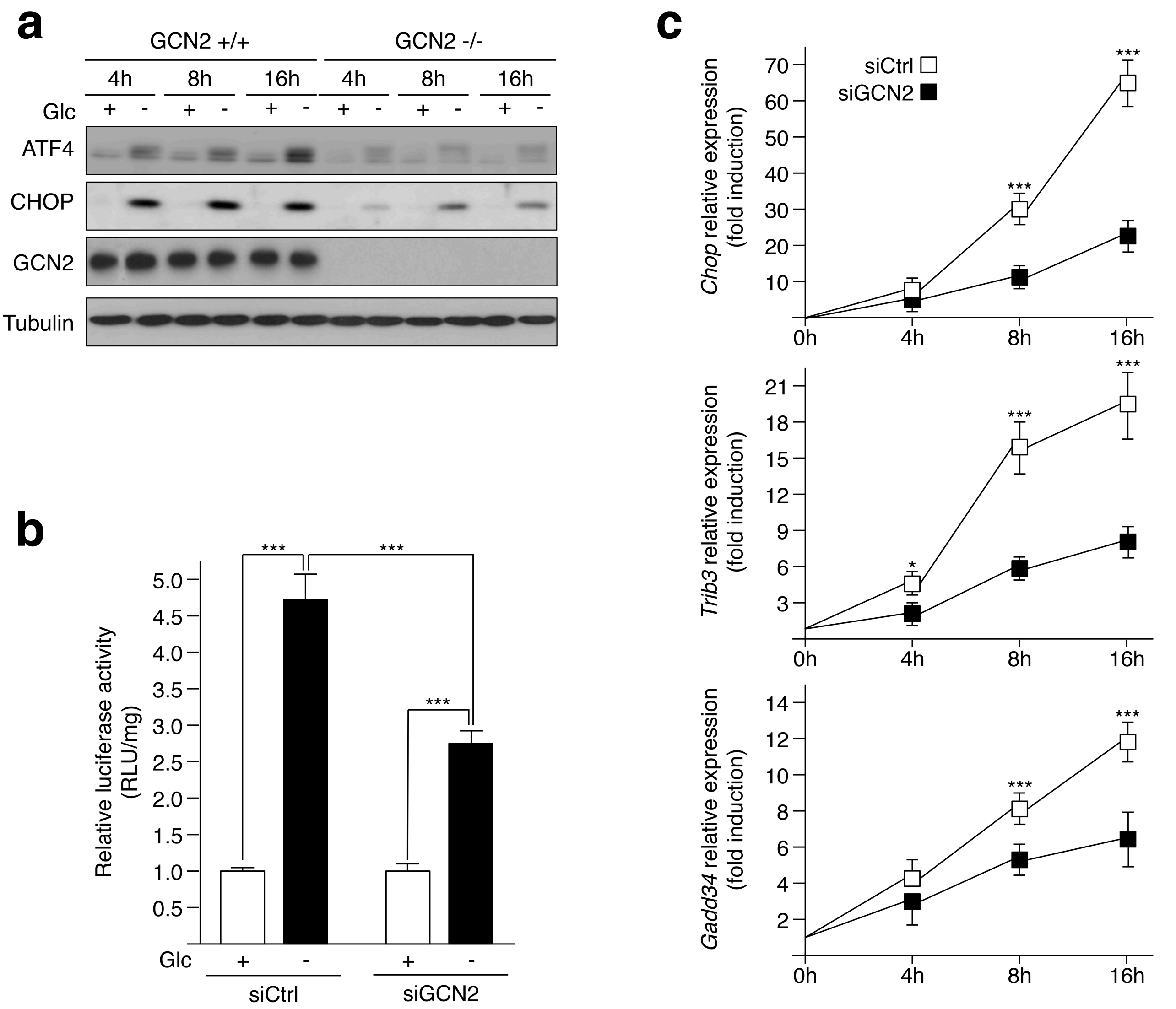

Supplementary Figure S4: GCN2 contributes to the ATF4-mediated transcription in response to glucose deprivation. (**a**) Immunoblot analysis of the abundance of ATF4, CHOP, and GCN2 in mouse embryonic fibroblasts wild type (+/+) or knockout (-/-) for GCN2 incubated in medium with 25 mM (+) or no (-) glucose for 4, 8 and 16 hours. Tubulin serves as a loading control. (**b**) Luciferase assays from HeLa cells stably expressing the ATF4 reporter system: CARE-Luc. Cells were prior transfected with control or GCN2 siRNAs. 72 hours following transfection, cells were incubated in a medium with 25 mM (+) or no (-) glucose. Data were normalized to the total protein content for each sample. *** $p < 0.001$. (**c**) RT-qPCR expression measurements of three ATF4-target model genes: *Chop*, *Trib3* and *Gadd34* in HBEC 3KT-RL transfected with control or GCN2 siRNAs and incubated for 4, 8 and 16 hours either in medium containing 25 mM (+) or 0.1 mM (-) of glucose. Data are expressed as fold inductions * $p < 0.05$ and *** $p < 0.001$.

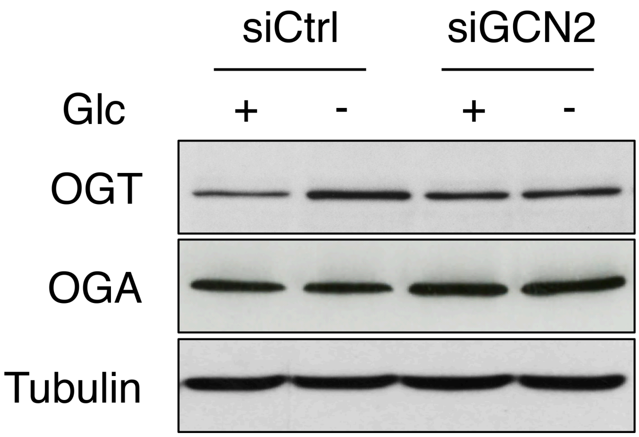

Supplementary Figure S5: OGT and OGA protein analysis in HBEC 3KT-RL cells silenced for GCN2 and glucose-deprived for 24 hours. Immunoblot analysis of the abundance of OGT and OGA, from HBEC 3KT-RL cells transfected with control or GCN2 siRNAs. Cells were incubated with DMEM containing 25 mM (+) or 0.1 mM (-) of glucose for 24 hours. Tubulin serves as a loading control.

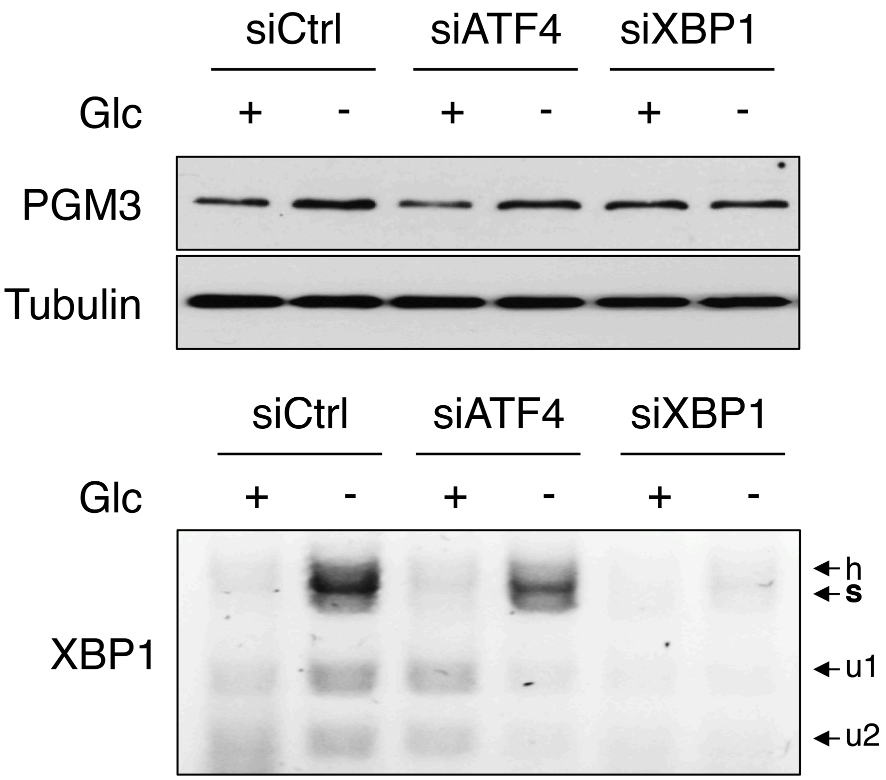

Supplementary Figure S6: PGM3 abundance in HBEC 3KT-RL cells silenced for ATF4 or XBP1 and glucose-deprived for 24 hours. Immunoblot analysis of the abundance of PGM3 and UAP1, from HBEC 3KT-RL cells transfected with control, ATF4 or XBP1 siRNAs. Cells were incubated with DMEM containing 25 mM (+) or 0.1 mM (-) of glucose for 24 hours. Tubulin serves as a loading control. Corresponding mRNAs were analyzed to assess XBP1 splicing by RT-PCR and Pst1 restriction: bold s- spliced XBP1, h- hydrid XBP1, u1 and u2- products from Pst1 cleavage of unspliced XBP1.

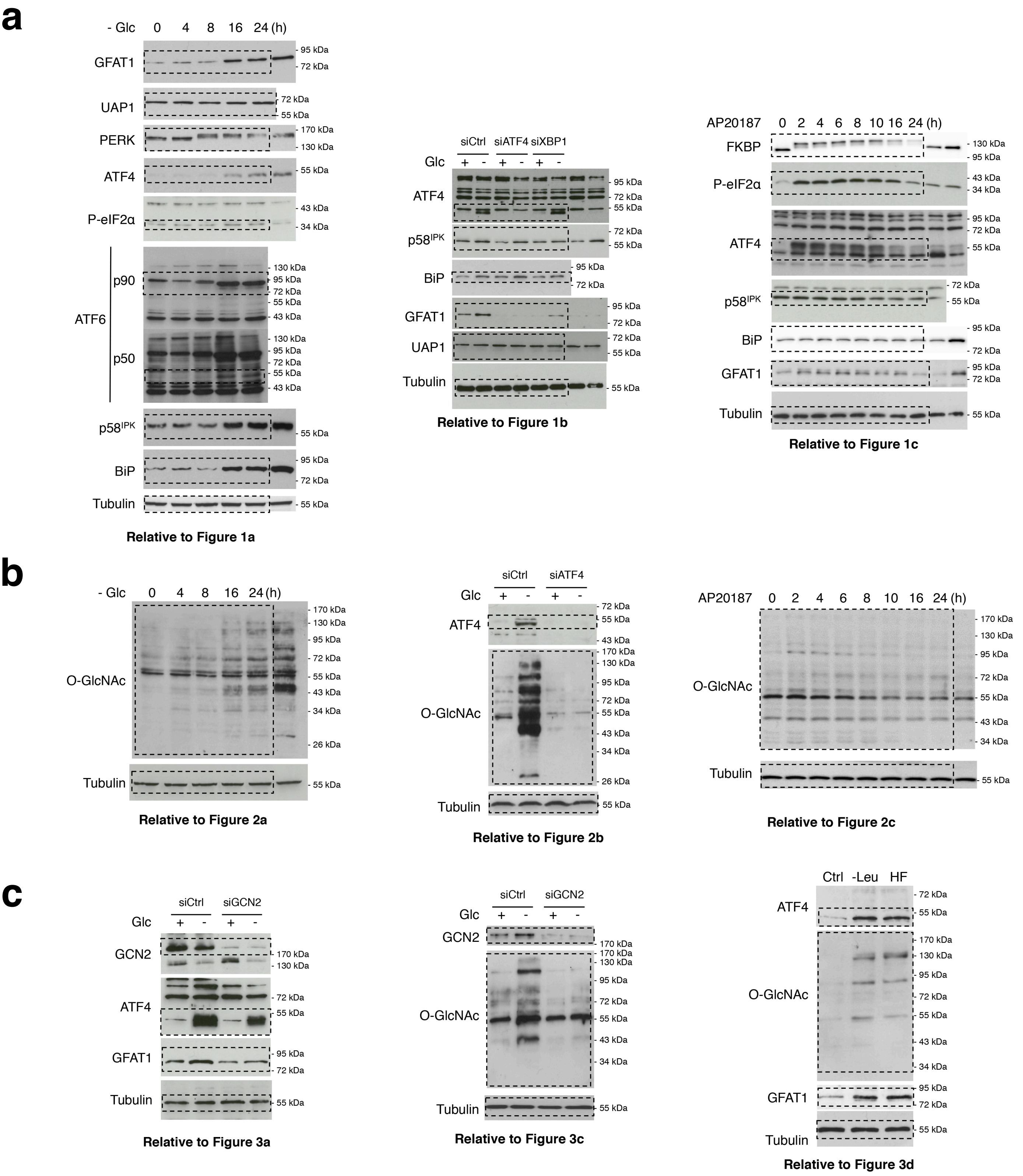

Supplementary Figure S7: Uncropped blots corresponding to main Figures 1, 2, and 3. All SDS-PAGE were run under the same experimental conditions. Following transfer, the membranes were generally cut into strips to minimize the required amount of antibody. Black boxes with dashed lines indicate how blots were cropped. (**a**) Uncropped blots for the images shown in Fig. 1a, 1b and 1c. (**b**) Uncropped blots for the images shown in Fig. 2a, 2b and 2c. (**c**) Uncropped blots for the images shown in Fig. 3a, 3c and 3d.

Human primers used for XBP1s RT-PCR

Gene name	Primer sequence
XBP1	Forward: AAACAGAGTAGCAGCTCAGACTGC<br>Reverse: TCCTTCTGGGTAGACCTCTGGGAG

Human RT-qPCR primers

Gene name	Primer sequence
Chop	Forward: AGCTGTGCCACTTTCCTTTC<br>Reverse: CAGAACCAGCAGAGGTCACA
Gadd34	Forward: CTGTGATCGCTTCTGGCA<br>Reverse: GGAAGAAAGGGTGGGCATC
Gfat1	Forward: CGGCTGCCTGATTTGATT<br>Reverse: GATAGCCTCGTCCCATTA
Hprt	Forward: TGACCTTGATTTATTTTGCATACC<br>Reverse: CGAGCAAGACGTTCAGTCCT
Trib3	Forward: TGGTACCCAGCTCCTCTACG<br>Reverse: GACAAAGCGACACAGCTTGA

Human primers used for ATF4 ChIP qPCR

Gene name	Primer sequence<br>Genome location of the amplicon
Gfat1	Forward: GTAATTCTCCTGCCTTGGCC<br>Reverse: GGTGTTTCATGTCCTGGGTG<br><br>Chr2:69318472+69318604

Human primers used for GFAT1 siRNA

Target	Primer sequence
Gfat1	Forward: GCAGAUACUUUGAUGGGUCUU<br>Reverse: AAGACCCAUCAAAGUAUCUGC

Supplementary Table S1: Primers sequences used for RT-PCR, RT-qPCR, ChIP experiments and GFAT1 siRNA.
